# Association between pulse pressure and carotid plaques in old adults with uncontrolled hypertension: results from a community-based screening in Hangzhou, China

**DOI:** 10.1186/s12872-024-03914-y

**Published:** 2024-05-11

**Authors:** Zhecong Yu, Haifeng Yang, Biqi Shou, Zongxue Cheng, Caixia Jiang, Jue Xu

**Affiliations:** 1 Hangzhou Center for Disease Control and Prevention, Hangzhou, 310000 P. R. China; 2 Fuyang Center for Disease Control and Prevention, Hangzhou, 311400 P. R. China; 3 Institute for Chronic Noncommunicable Disease Control and Prevention, Hangzhou Center for Disease Control and Prevention, Hangzhou, 310000 P. R. China

**Keywords:** Carotid plaques, Pulse pressure, Old adults, Uncontrolled hypertension, Community screening

## Abstract

**Background:**

There is a broad pulse pressure (PP) and a high prevalence of carotid plaques in old adults. Previous studies have indicated that PP is strongly associated with carotid plaque formation. This study aimed to explore this association in old adults with uncontrolled hypertension.

**Methods:**

1371 hypertensive patients aged ≥ 60 years with uncontrolled hypertension were enrolled in a community-based screening in Hangzhou, China. Carotid plaques were assessed using ultrasonography. Logistic regression models were used to estimate the association between PP and carotid plaques by odds ratios (ORs) and 95% confidence intervals (CIs).

**Results:**

Carotid plaques were detected in 639 (46.6%) of subjects. Multiple plaques were found in 408 (63.8%) and soft plaques in 218 (34.1%). Elevated PP was associated with a high prevalence of carotid plaques. After adjusting for traditional risk factors, compared to patients within the lowest tertile of PP, those within the highest tertiles had an increased risk of carotid plaques (OR 2.061, CI 1.547–2.745). For each 1-SD increase, the risk increased by 40.1% (OR 1.401, CI 1.237–1.587). There was a nonlinear association between PP and carotid plaques (P nonlinearity = 0.039). The risk increased rapidly after the predicted PP level reached around 60 mmHg. The associations were stronger among participants with multiple and soft plaques.

**Conclusions:**

Our findings suggested that PP was independently associated with carotid plaques in old adults with uncontrolled hypertension who have an increased risk of atherosclerosis.

**Supplementary Information:**

The online version contains supplementary material available at 10.1186/s12872-024-03914-y.

## Introduction

Hypertension is a major public health challenge in China, and its prevalence is rising, but it has not been sufficiently controlled. Nearly 50% of Chinese adults aged 35–75 years have hypertension, while only less than 10% control it [1]. It is well known that hypertension is the leading cause of cardiovascular death. Compared to hypertensive patients with controlled blood pressure (BP), those who fail to control BP have a higher risk of cardiovascular death [2, 3]. This adverse outcome risk increases with increasing age. China follows the global trend of population aging [4], and regulating BP in the senior age group is not ideal [1]. Therefore, the aging group with uncontrolled hypertension deserve more attention in daily healthcare.

Carotid plaque formation is a common risk factor for cardiovascular and cerebrovascular diseases such as stroke, transient ischemic attack (TIA), and acute myocardial infarction [5–7]. About 31% of the Chinese population have carotid plaques, and up to 63% of the old adults aged 70–89 years [8]. In 2020, ∼ 200 million people in China were affected by carotid plaques [9]. It was necessary to take timely intervention to prevent carotid atherosclerosis. Pulse pressure (PP) was strongly associated with carotid plaques. Age-related central elastic arteriosclerosis and high systolic blood pressure (SBP) make old adults more likely to have a wide PP [10]. Previous studies in China showed that elevated PP was an independent risk factor for the presence of carotid plaques [11–14]. However, there is limited information about this correlation in old adults with uncontrolled hypertension, who are more vulnerable to atherosclerosis diseases.

Therefore, this study aimed to find the association between PP and carotid plaques in old adults with uncontrolled hypertension. We also investigated the dose-response relationship between PP levels and carotid plaques, independent of the traditional risk factors.

## Methods

### Study design and participants

**Fig. 1:**
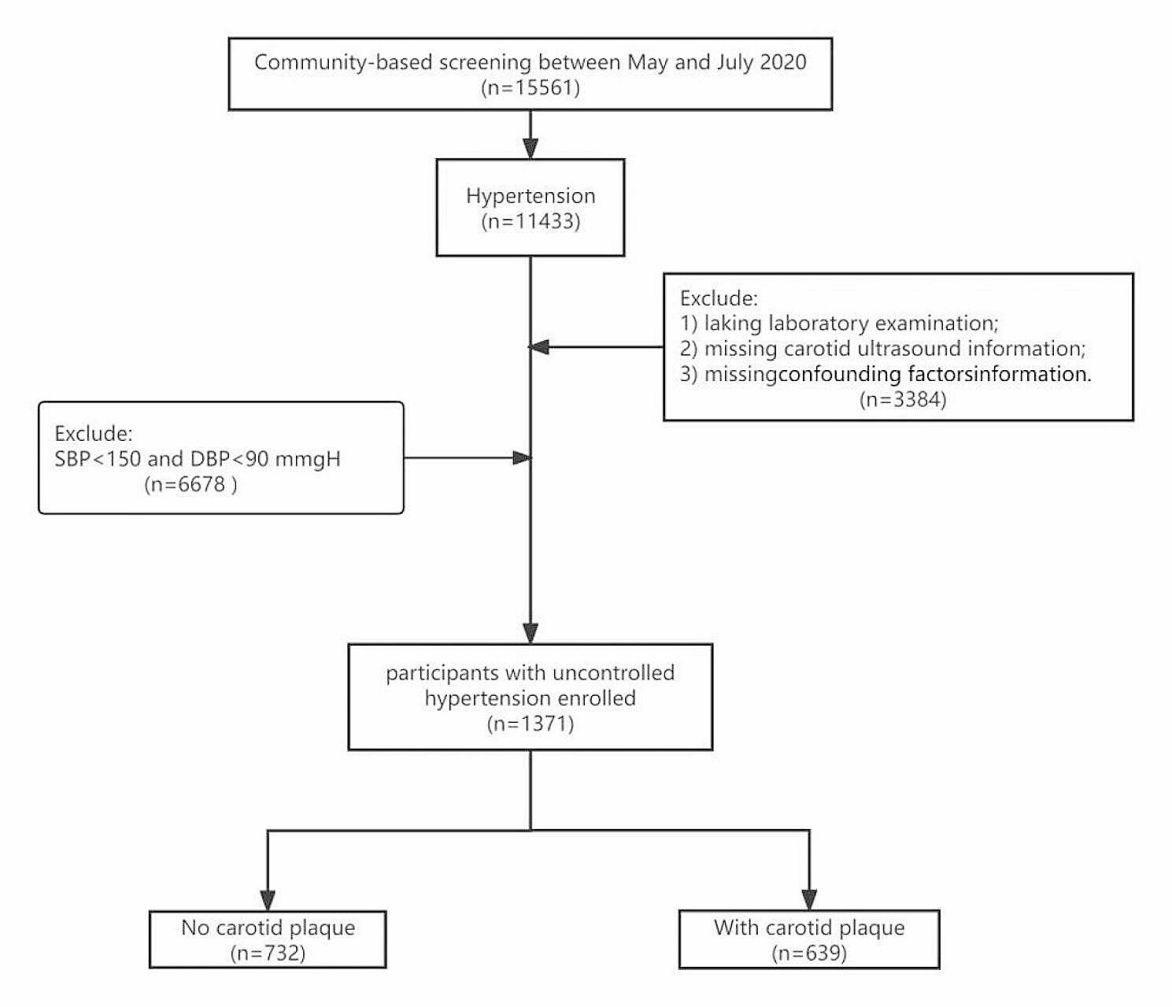
Flowchart of the study

In May 2020, the Centers for Disease Control and Prevention and the medical service community collaborated on a “stroke high-risk population screening and intervention” initiative in Hangzhou, China. This was a large-scale public welfare project that focused on secondary prevention and individualized intervention for high-risk population of stroke. Participants were informed of the screening results by community health workers, and further diagnosis and treatment would be recommended if necessary. Participants aged ≥ 60 years with hypertension or diabetes were selected from the service contractor of the family doctor of their community health service centre, and they were informed of the screening details, including a questionnaire survey, physical examination, and carotid ultrasonography, by their family doctor. Participants meeting the following criteria were excluded from the screening: (1) Patients with mental disorders are unable to complete the questionnaire independently; (2) Long-term disability; (3) Unable to complete the follow-up. This screening included a sample of 15,561 participants according to the above exclusion criteria. For the present study, of the 11,433 participants with hypertension who were included, 3384 participants had incomplete information on biochemical indexes, conventional confounding factors or carotid artery imaging and 6678 participants were excluded due to having SBP < 150 and diastolic blood pressure (DBP) < 90 mmHg. Finally, 1371 patients were included in this analysis (Fig. 1).

### Data collection

Detailed information on individual demographics, lifestyle, and histories of disease was obtained through standardized self-report questionnaires. Smoking was defined as those who had been continuously or cumulatively smoking for ≥ 6 months. Regular exercise was defined as moderate/vigorous-intensity performed ≥ 30 min 3 or more times per week. Physical examinations include height, weight, SBP, and DBP. Height was measured in a standing position, and weight was measured using a calibrated platform scale. Participants completed the above measurement process in a light dress and without shoes. Body mass index (BMI) was calculated as weight/ height^2^ (kg/ m^2^). BP measurements were determined by the participants sitting in a quiet room at their local community health center. Participants were instructed to avoid alcohol consumption, cigarette smoking, coffee/tea, and exercise for at least 30 min before BP measurement. BP was measured using an automatic electronic sphygmomanometer (HEM-741 C; Omron, Tokyo, Japan). For each participant, the BP was measured 3 times by community health workers, and the average of three BP was used for the final analysis and evaluation. For the current participants (age ≥ 60 years), based on the Eighth Joint National Committee [15], uncontrolled hypertension for the present analysis was identified if the SBP was ≥ 150 mmHg or the DBP was ≥ 90 mmHg, regardless of antihypertensive medications use (Supplemental Fig. 1). Biochemical measurements include blood glucose, creatinine and lipid levels, including triglyceride (TG), total cholesterol (TC), low density lipoprotein cholesterol (LDLC), and high density lipoprotein cholesterol (HDLC). Blood samples were drawn from each participant’s antecubital vein in the morning after an overnight fast. Fasting blood glucose, TG, and TC were measured using the glucose oxidase method, glycerol phosphate oxidase-peroxidase, and esterase oxidase-peroxidase, respectively. HDLC was determined after phosphotungstate precipitation. LDLC was calculated using the Friedewald formula [16]. Atherogenic dyslipidemia was defined as TG > 1.69 mmol/L and HDLC < 1.03 mmol/L in males or < 1.29 mmol/L in females [17]. The estimated glomerular filtration rate (eGFR) was calculated as follows: eGFR = 175× Scr^− 1.234^× age^− 0.179^ (if female,×0.79), where Scr is serum creatinine concentration (in mg/dL) and age in years. Reduced renal function was defined as eGFR < 60 mL/min per 1.73 m² [18].

Carotid ultrasonography examinations were certified manually by experienced sonographers who received unified training from the local medical service community and were blinded to the characteristics and laboratory results of the participants. All participants were studied lying supine with their head turned 45 degrees from the site being scanned. The colour Doppler ultrasound diagnostic instrument was equipped with a Philips HDI 5000 ultrasound system and a 7.5 MHz probe, which was operated by sonographers. We examined the carotid plaques on the left and right sides, with each side measured at three different locations: common carotid artery, carotid bifurcation, and internal carotid artery. Carotid plaques were defined as focal structures encroaching into the arterial lumen of at least 0.5 mm or 50% of the surrounding intima-media thickness value or demonstrating a thickness > 1.5 mm as measured from the intima-lumen interface to the media-adventitia interface [19].

### Statistical analysis

Demographic and clinical characteristics were expressed as mean ± standard deviation (SD) for continuous variables and proportions with corresponding percentages (%) for categorical and binary variables. The preliminary analysis explored differences in characteristics of participants by uncontrolled hypertension according to PP levels, using ANOVA or Kruskal-Wallis tests for continuous variables, while for nominal variables, chi-square tests were performed, as appropriate. Participants were grouped into tertiles according to the PP levels: T3, ≥ 79 mmHg; T2, 65–79 mmHg; T1, < 65 mmHg. Binary logistic regressions were conducted to assess the association between PP levels and carotid plaques and to calculate the odds ratios (ORs) and 95% confidence intervals (CIs). A test for multicollinearity would be conducted using an ordinary least squares (OLS) model before analysis. No violation was found as all the variables displayed a variance inflation factor (VIF) < 5. Two models were used. Model 1 was unadjusted. In model 2, we adjusted for a propensity score. The propensity score was calculated with a linear regression model entering PP as the dependent variable. The independent variables included sex, age, smoking, regular exercise, history of stroke, history of transient ischemic attacks (TIA), history of atrial fibrillation/valvular heart disease, diabetes, family history of stroke, BMI, blood glucose, TC, TG, LDLC, HDLC, eGFR, antihypertensive medications and lipid-lowing medications. In the above models, PP was entered as a categorical variable with T1 as the reference group or as a continuous variable with per 1-SD increment. Restricted cubic splines were performed with four knots at the 5th, 33.3th, 50th, 66.6th, and 95th centiles to flexibly model the association between PP and the risk of carotid plaques with adjusted model 2. We also assessed the relationship between PP and the risk of multiple plaques (plaques ≥ 2) and plaque type, including soft, hard and mixed plaques, by using multinomial Logistic regression. In addition, the risk of carotid plaques by categories of age and PP (grouped by the median) would be evaluated. Subgroup analyses were carried out using multivariable Logistic models stratified by traditional clinical risk factors, including sex, smoking, BMI, BP, atherogenic dyslipidemia and reduced renal function, and were summarized via forest plots. An interaction term within each subgroup was also included using the likelihood ratio test. Finally, for sensitivity analysis, we excluded patients with a history of stroke, TIA, taking lipid-lowering or not taking antihypertensive medications and used Logistic models to repeat the analysis to verify the robustness of the results.

Data analysis was performed using the SPSS 25.0 software (SPSS Inc., Chicago, IL, USA) and SAS 9.4 software (SAS Institute Inc., Cary, NC, USA). All statistical tests were two-tailed, with *P* < 0.05 considered statistically significant.

## Results

**Fig. 2:**
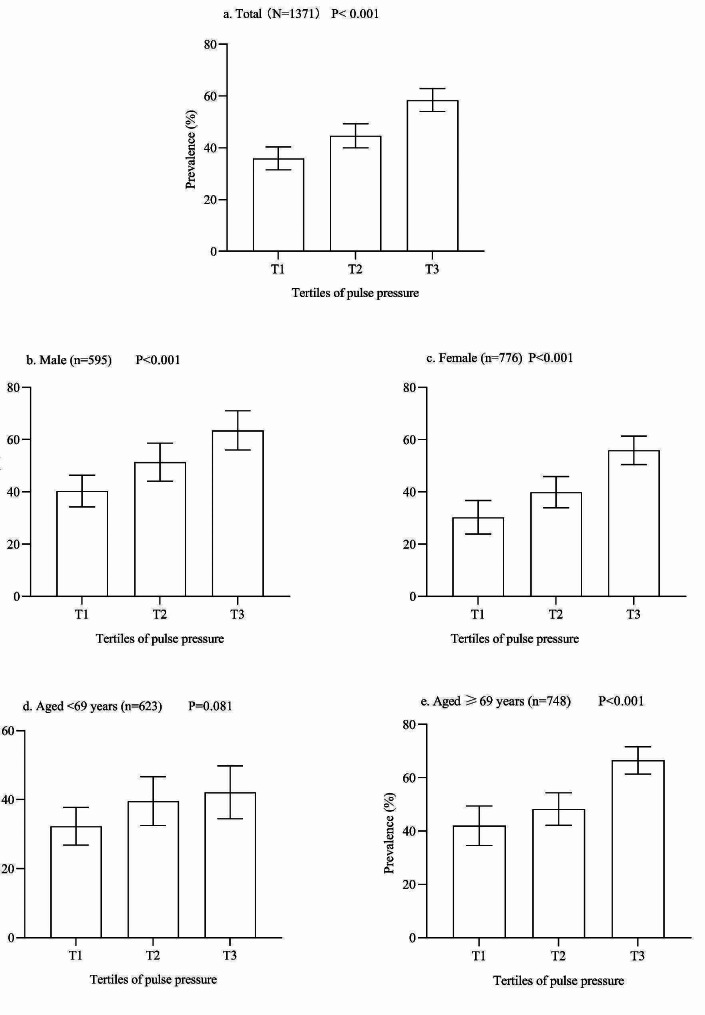
Bar graphs showing the prevalence of carotid plaque according to pulse pressure tertiles. The error bars indicated the confidence interval. Age was grouped as the median

The characteristics of the study population according to tertiles of PP were presented in Table 1. Among the studied 1371 participants, the mean age was 70.21 ± 5.99 years, and 776 (56.60%) were female. Participants in the highest tertiles of PP were more likely to be female, relatively older and have a higher SBP. The proportions of family history of stroke and smoking were more frequent in participants with the lowest tertiles of PP. The median PP was 71 mmHg (interquartile range, 60–81 mmHg). As shown in Fig. 2, the prevalence of carotid plaques increased with the increase in PP levels, regardless of sex. Higher prevalence was more often observed in older groups. A statistical difference in the distribution of carotid plaques prevalence on PP tertiles was found only among participants with SBP ≥ 150 mmHg and DBP < 90 mmHg (Supplemental Fig. 2).

**Table 1:** Characteristics of patients with uncontrolled hypertension by pulse pressure tertiles

Characteristics	Total, *n* = 1371	T1(< 65 mmHg), *n* = 451	T2 (65–79 mmHg), *n* = 441	T3(≥ 79 mmHg), *n* = 479	*P*-value
Age, years	70.21 ± 5.99	67.75 ± 4.89	70.41 ± 5.45	72.35 ± 6.52	< 0.001
Female, n (%)	776(56.60)	198(43.90)	258(58.50)	320(66.81)	< 0.001
Smoking, n (%)	210(15.32)	85(18.85)	74(16.78)	51(10.65)	0.001
Regular exercise, n (%)	464(33.84)	136(30.16)	152(34.47)	176(36.74)	0.099
History of stroke, n (%)	86(6.27)	28(6.21)	24(5.44)	34(7.10)	0.584
History of TIA, n (%)	96(7.00)	37(8.20)	26(5.90)	33(6.89)	0.938
Family history of stroke, n (%)	185(13.49)	74(16.41)	59(13.38)	52(10.86)	0.046
Diabetes, n (%)	328(23.92)	72(15.96)	111(25.17)	145(30.27)	< 0.001
Atrial fibrillation /valvular heart disease, n (%)	181(13.20)	48(10.64)	59(13.38)	74(15.45)	0.095
Blood glucose, mmol/L	6.12 ± 1.67	5.82 ± 1.37	6.27 ± 1.92	6.27 ± 1.66	< 0.001
TC, mmol/L	4.82 ± 1.07	4.79 ± 1.08	4.77 ± 1.12	4.91 ± 1.06	0.118
TG, mmol/L	1.88 ± 1.34	1.88 ± 1.45	1.89 ± 1.29	1.86 ± 1.29	0.906
HDLC, mmol/L	1.39 ± 0.35	1.39 ± 0.34	1.38 ± 0.35	1.42 ± 0.37	0.219
LDLC, mmol/L	2.71 ± 0.80	2.65 ± 0.78	2.69 ± 0.80	2.77 ± 0.81	0.067
eGFR, mL/min per 1.73m^2^	64.80 ± 43.34	65.98 ± 28.93	63.04 ± 41.63	65.29 ± 54.74	0.573
BMI, kg/m2	19.63 ± 2.82	20.24 ± 2.56	19.34 ± 2.68	19.31 ± 3.07	< 0.001
SBP, mm Hg	154.89 ± 13.67	143.04 ± 10.56	155.73 ± 6.10	165.26 ± 12.46	< 0.001
DBP, mm Hg	84.44 ± 10.27	92.89 ± 6.82	84.37 ± 6.49	76.56 ± 9.47	< 0.001
Antihypertensive medications					
ACEI, n (%)	1355(98.83)	448(99.33)	438(99.32)	469(97.91)	0.067
ARB, n (%)	714(52.08)	259(57.43)	212(48.07)	243(50.73)	0.015
β-blockers, n (%)	71(5.18)	25(5.54)	20(4.54)	26(5.43)	0.758
CCB, n (%)	777(56.67)	253(56.10)	246(55.78)	278(58.04)	0.753
Diuretics, n (%)	339(24.73)	127(28.16)	106(32.17)	106(34.94)	0.095
Lipid-lowering medications, n (%)	5(0.36)	2(0.44)	1(0.23)	2(0.42)	0.842

Data were shown as mean ± SD for continuous variables and n (%) for categorical variables. BMI, body mass index; TIA, transient ischemic attacks; SBP, systolic blood pressure; DBP, diastolic blood pressure; TC, total cholesterol; TG, triglycerides; HDLC, high density lipoprotein cholesterol; LDLC low density lipoprotein cholesterol; eGFR estimated glomerular filtration rate; ACEI angiotensin-converting enzyme inhibitor; ARB angiotensin receptor blocker; CCB calcium channel blocker

In Tables 2, 639 (46.6%) participants were found to have carotid plaques. Logistic regression analyses showed significant associations between PP and carotid plaques. In the crude model (**model 1)**, compared with the participant with PP < 65 mmHg (T1), 65–79 mmHg (T2) and ≥ 79 mmHg (T3) both had a higher risk of carotid plaques [unadjusted odds ratio (OR): 1.440; 95% confidence interval (CI): 1.010–1.884 and OR: 2.510; 95% CI: 1.926–3.270, respectively.] After fully adjusted (**model 2**), compared with the T1 group, T3 remained a positive association with the risk of carotid plaques (OR: 2.061; 95% CI: 1.547–2.745), no significant differences were observed in the T2 group (OR: 1.269; 95% CI: 0.960–1.677). When considering PP as per 1-SD mmHg, there was also a positive association in both models. As shown in Tables 3 and 408 (63.8%) of participants with carotid plaques have multiple plaques. Compared with the T1 group, T3 increased by 136.6% risk of having multiple plaques than no plaques (OR: 2.366; 95% CI: 1.700–3.293). In addition, 218 (34.1%) have soft plaques. Compared with the T1 group, T3 increased by 208.5% risk of having soft plaques than no plaques (OR: 3.085; 95% CI: 1.423–6.687).

**Table 2:** The relationship between pulse pressure levels and carotid plaque

ORs (95% CI)	Tertiles of PP			PP, per 1-SD mmHg
	T1	T2	T3	
Model 1	Ref.	1.440 (1.101–1.884)	2.510 (1.926–3.270)	1.517 (1.352–1.701)
Model 2	Ref.	1.269 (0.960–1.677)	2.061 (1.547–2.745)	1.401 (1.237–1.587)

Model 1: unadjusted, Model 2: adjusted for a propensity score. OR odds ratio; CI confidence interval; PP pulse pressure

**Table 3:** Multinomial Logistic regression with OR (Cls) for plaques number and type

Outcomes	Tertiles of PP			PP, per 1-SD mmHg
	T1	T2	T3	
Plaques number				
Multiple plaques vs. One plaque	Ref.	0.926 (0.599–1.432)	1.477 (0.954–2.287)	1.227 (1.019–1.477)
Multiple plaques vs. No plaques	Ref.	1.237 (0.886–1.726)	2.366 (1.700–3.293)	1.517 (1.313–1.753)
One plaque vs. No plaques	Ref.	1.335 (0.916–1.946)	1.602 (1.076–2.384)	1.236 (1.045–1.462)
Plaques type				
Mixed^※^ vs. No plaques	Ref.	1.308 (0.934–1.832)	2.194 (1.562–3.082)	1.467 (1.267–1.700)
Soft vs. No plaques	Ref.	1.793 (0.822–3.910)	3.085 (1.423–6.687)	1.636 (1.186–2.257)
Hard vs. No plaques	Ref.	1.113 (0.744–1.664)	1.684 (1.122–2.527)	1.252 (1.053–1.489)
Soft vs. Hard	Ref.	1.611 (0.695–3.738)	1.832 (0.799–4.200)	1.307 (0.926–1.845)

Multinomial Logistic regression model was adjusted as model 2 in Table 2. OR odds ratio; CI confidence interval; PP pulse pressure; ^※^ Included individuals with hard and soft plaques in bilateral carotid arteries

**Fig. 3:**
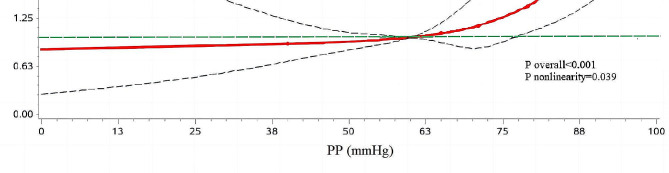
Dose-response relationship between pulse pressure levels and the risk of carotid plaque. Red lines indicated hazard ratios, the green line indicated the reference line (the median of pulse pressure as a reference point), and the dashed lines indicated 95% CI from restricted cubic spline regression. ORs were calculated using Logistic regression analysis after adjustments for a propensity score. OR odds ratio; CI confidence interval

As shown in Fig. 3, Logistic regression with restricted cubic splines was conducted to model and visualize the association between predicted PP and carotid plaques. When considering the PP at 60 mmHg as the reference point [20], the risk of carotid plaques increased slowly until 60 mmHg of predicted PP and then started to grow rapidly (P nonlinearity = 0.039). There was a substantial increase in the associated risk after 60 mmHg.

**Fig. 4:**
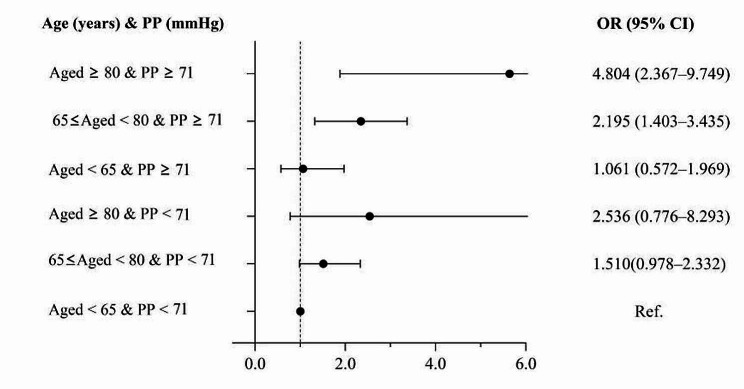
To assess the risk of carotid plaque by grouping of interactions of age and PP. Data were adjusted for a propensity score. OR odds ratio; CI confidence interval; PP pulse pressure

In Fig. 4, we further examined the association of carotid plaques based on categories of age and PP. The increase in age and PP were associated with the risk of carotid plaques. Compared to the participants with aged < 65 years and PP < 71 mmHg, those aged 80 ≥ years and PP ≥ 71 mmHg had the highest risk of carotid plaques (OR: 4.804; 95% CI: 2.367–9.749).

**Fig. 5:**
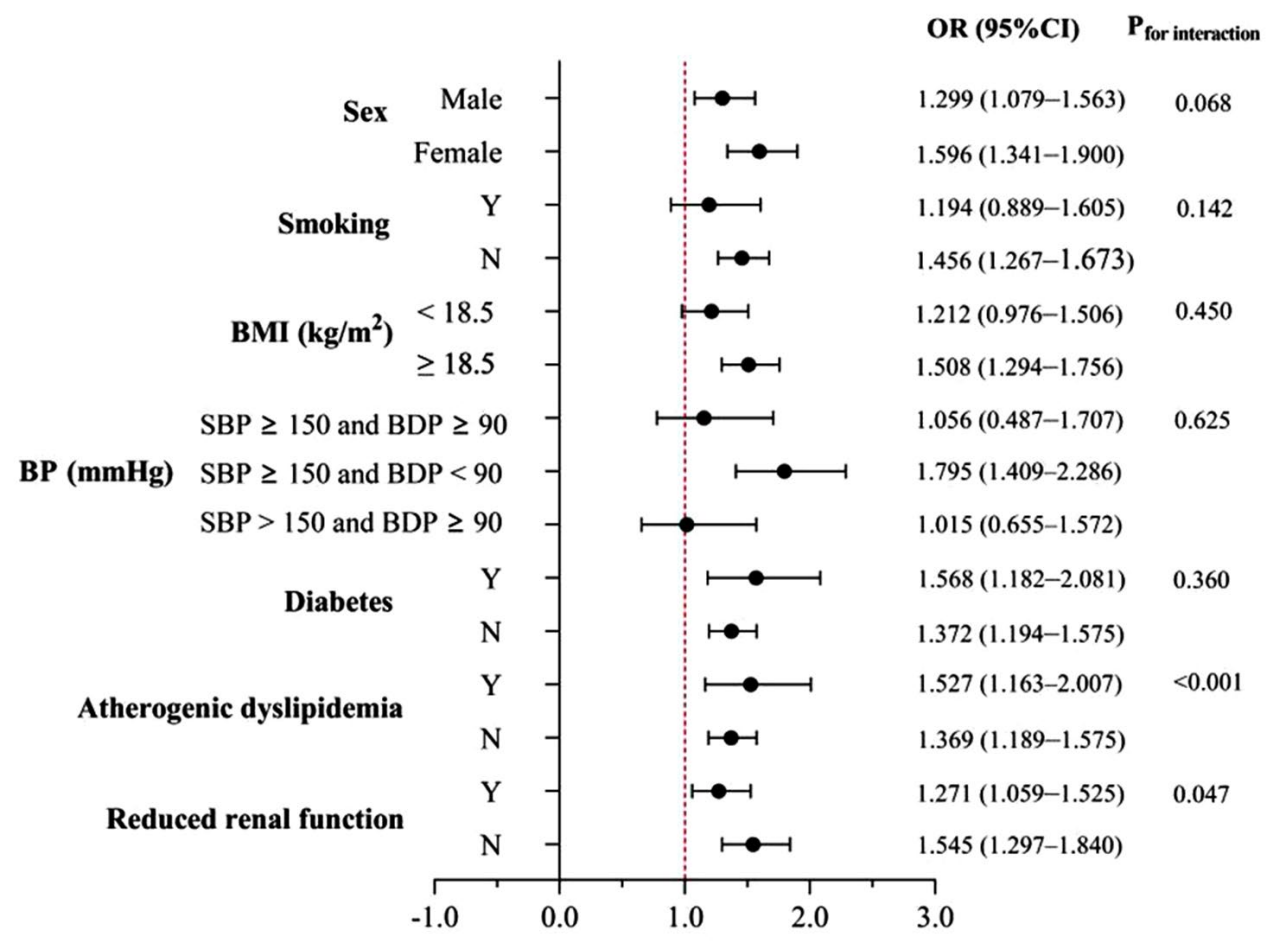
Associations between pulse pressure and the risk of carotid plaque in patients stratified by the sex, BMI, smoking, blood pressure, diabetes, atherogenic dyslipidemia and reduced renal function. Data were adjusted for a propensity score other than variables for stratification. ORs per 1-SD mmHg increase in pulse pressure. The P-value for interaction was calculated. OR odds ratio; CI confidence interval. Other abbreviations as in Table 1

Figure 5 presented the carotid plaques for the PP (per 1-SD mmHg) in various subgroups. The association between PP levels and carotid plaques risk was stronger in atherogenic dyslipidemia and participants without reduced renal function. There were no significant interactions of sex, smoking, BMI, BP groups and diabetes.

In the sensitivity analysis, participants with a history of stroke, TIA, taking lipid-lowering or not taking antihypertensive medications were excluded. PP (per 1-SD mmHg or grouped by tertiles) was still related to a higher risk of carotid plaques after adjusting for multiple covariates (Supplemental Table 1).

## Discussion

In this cross-sectional study, we used 1371 community-dwelling old adults with uncontrolled hypertension aged ≥ 60 years to evaluate the association between PP and carotid plaques. The results revealed a positive association between PP and the risk of carotid plaques. We found that the risk of carotid plaques significantly increased with PP levels after the inflection point (60 mmHg). The association between the PP and carotid plaques was closer among patients with multiple and soft carotid plaques. Our results shed light on the association between PP and the risk of carotid plaques and tried to provide a potential threshold for intervention to reduce the arteriosclerosis burden in old adults with uncontrolled hypertension.

Previous results of studies on the relationship between PP and carotid plaques risk in old adults or hypertensive patients have been limited and challenging to replicate. Consistent with our results, Dong et al. found that elevated PP was an independent risk factor for carotid plaques in participants aged ≥ 60 years without histories of stroke or cardiovascular disease in rural China [11]. PP was also associated with stenosis of the asymptomatic carotid artery and angiographic ulceration of the symptomatic carotid artery [21], as well as promoting an increase in plaque number [12], independently of antihypertensive medications use. Among old adults with uncontrolled hypertension, PP was strongly associated with multiple plaques and soft plaques. Soft plaques, also called vulnerable plaques, are more likely to be unstable and dislodged. In 323 hypertensive patients with a mean age of 61.7 ± 14 years, high SBP and low DBP were associated with uncontrolled hypertension. High PP was associated with increased common artery intima-media thickness but not with carotid or iliofemoral plaques [22]. However, the present study only evaluated the association between PP and carotid plaques, its number and type, but not with plaque in other sites, such as coronary, iliofemoral intracranial or extracranial arteries, and common artery intima-media thickness size. These inevitably limited the comparison of our findings with others. In addition, participants in the above study were recruited from the Department of Cardiology, and at least 30% were diagnosed with cardiovascular disease. In contrast, 1355 (98.83%) of the community residents with uncontrolled hypertension taking at least one antihypertensive medication were enrolled, and the positive association remained significant in participants without a history of stroke or TIA. A longitudinal study from Norway found that PP could not effectively predict plaque burden, echolucent plaques, and carotid intima-media thickness. A single time-point measurement of PP at age 40 was not associated with atherosclerosis two decades later [23]. The effects of PP and its dynamic changes on atherosclerosis need to be further studied in essential/ secondary hypertensive patients. Therefore, generalizability or potential selection bias should be considered when interpreting our results.

This study found a cut-off value for the association between PP and carotid plaques. Previous studies showed that both cardiac target organ damage and cardiovascular prognosis were related to increased PP, suggesting a specific cut-off (> 60 mmHg) for participants aged ≥ 60 years [24, 25]. Another large longitudinal cohort study of patients with a high risk of atherosclerosis indicated a PP ≥ 70 mmHg increased risk of cardiovascular events [26]. The results of the study on high-risk participants were consistent with the results we reported. Previous studies indicated that PP and SBP were superior to DBP in predicting intima-media thickening and early plaques [27]. High PP levels could induce endothelial dysfunction to promote carotid plaques [28]. However, PP may be a maker of widespread atherosclerosis rather than a cause, whereas stiffness of the arterial tree leads to an increase in SBP and a decrease in DBP [29]. In addition, aging was closely related to high PP, mainly due to age-related alterations in collagen to elastin ratio and disruption of elastic fibres [30]. Increased PP was associated with the markers of subclinical cardiovascular disease, and this association was stronger with aging in a cohort study [31]. Our results found that advanced age and high PP were closely related to the risk of carotid plaques. After the age of 60 years, DBP decreased with age, while SBP increased [32]. Isolated systolic hypertension was the primary type of hypertension in old adults [33]. However, the absence of a significant interaction between hypertension type and PP with carotid plaques may reflect a lack of statistical power to detect effect in this not large sample, despite the association between PP and carotid plaques being larger among those with SDP ≥ 150 and DBP < 90 mmHg than those with SDP < 150 and DBP ≥ 90 mmHg. In cross-sectional studies of patients with kidney disease [34, 35], PP was weakly associated with large vessel calcification and atherosclerosis. The authors indicated that the influence of hypervolemia may overwhelm the impact of atherosclerosis and arterial calcification on PP. Patients with chronic kidney disease tend to have retention of water and sodium, which led to blood volume expansion [36]. Consistently, our results found that the association between PP and carotid plaques risk was weaker in participants with reduced renal function.

The main strength of this study was that we presented a general description of carotid plaques prevalence in old adults with uncontrolled hypertension and comprehensively investigated the associations between PP and the risk of carotid plaques from a community screening. To our knowledge, this association has not previously been reported in detail in such a high-risk group. However, several limitations should be noted here. First, this study was a cross-sectional study and was not randomized. The ability to clarify a cause-effect of PP and carotid plaques in old adults with uncontrolled hypertension was limited. Therefore, it is necessary to continue to record the BP and the occurrence of atherosclerosis in these old adults. Second, this study was restricted to a not large urban sample in southeast China, which is a potential limiting factor to the generalization of our results under the social background of aging and poor blood pressure control. Third, carotid artery imaging results were measured by one certified sonographer. Apart from the fact that we could not assess inter-rater variability for the measurements, residual bias from diagnostic suspicion was required to be considered. Finally, we did not collect some potential confounders, such as inflammatory biomarkers, dietary habits, and herb use. The absence of these data may compromise the validity of the present results.

## Conclusions

In conclusion, in a community screening of old adults with uncontrolled hypertension, an elevated PP was associated with the risk of carotid plaques as well as promoting multiple plaques and unstable plaques, independently of lifestyle characteristics and other cardiovascular risk factors. Our findings may guide community health workers to help old adults control the PP at an optimal level to reduce the risk of carotid plaques. Future prospective studies with a large sample are necessary to verify our findings and to provide a more precise intervention threshold of PP in old adults with uncontrolled hypertension.

### Electronic supplementary material

Below is the link to the electronic supplementary material.

Supplementary Material 1

## Data Availability

The data sets used during the current study are available from the corresponding author on reasonable request.

## Abbreviations

TIA: Transient ischemic attack
PP: Pulse pressure
SBP: Systolic blood pressure
DBP: Diastolic blood pressure
BMI: Body mass index
TC: Total cholesterol
LDLC: Low density lipoprotein cholesterol
HDLC: High density lipoprotein cholesterol
SD: Standard deviation
OR: Odds ratio
CI: Confidence interval
OLS: Ordinary least squares
VIF: Variance inflation factor
