# Supplementary material for: Association between pulse pressure and carotid plaques in old adults with uncontrolled hypertension: results from a community-based screening in Hangzhou, China
Source: BMC Cardiovasc Disord. 2024 May 11;24:249. doi: 10.1186/s12872-024-03914-y (PMC11088081; doi:10.1186/s12872-024-03914-y)
Supplement: Supplementary file 1 — Supplementary Material 1 [file 12872_2024_3914_MOESM1_ESM.docx]

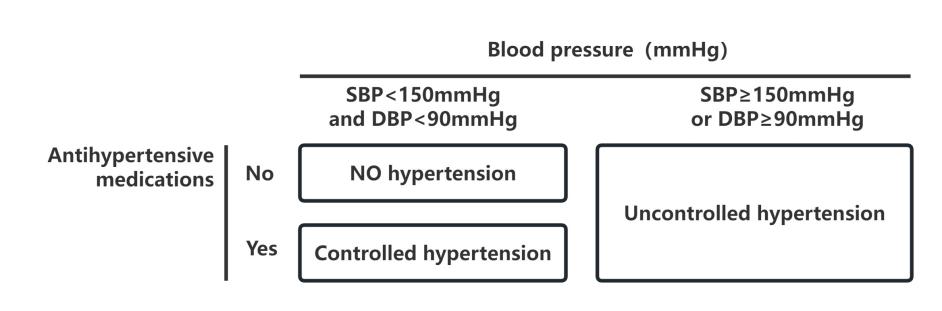


**Supplemental Figure 1** Definition of categories of hypertension control. SBP systolic blood pressure; DBP Diastolic Blood Pressure.


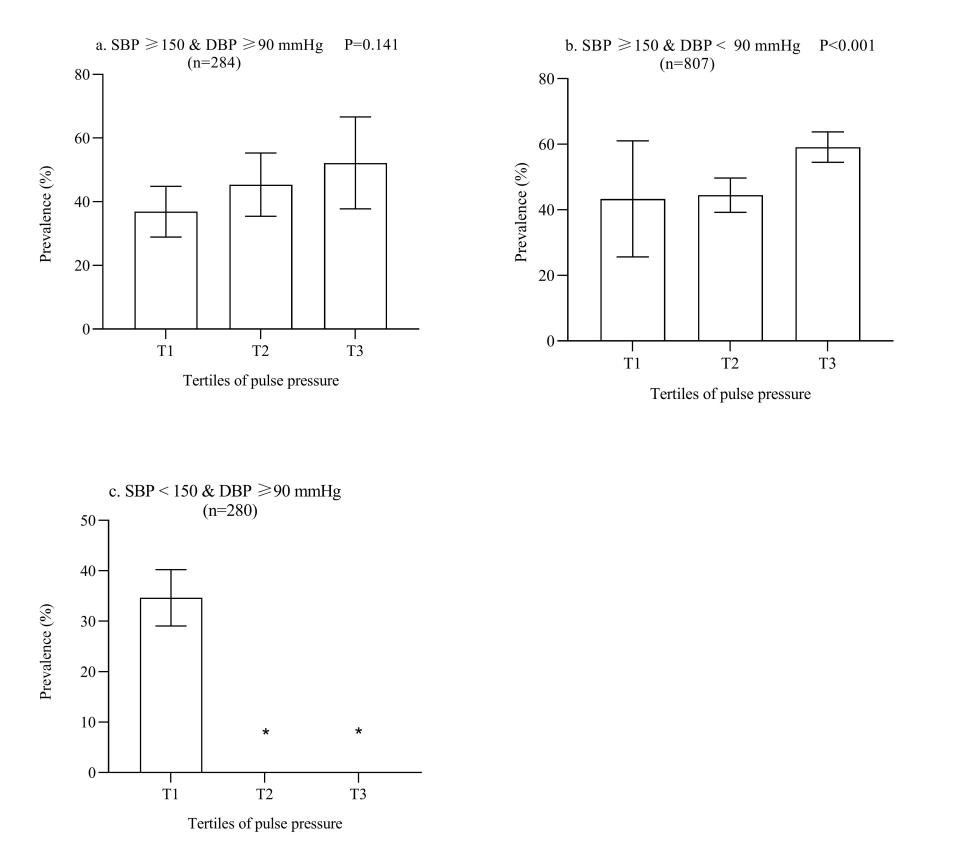


**Supplemental Figure 2** Bar graphs showing the prevalence of carotid plaque according to pulse pressure tertiles in hypertension types. The error bars indicate the confidence interval. Age was grouped as the median. * No observations.

| **Supplemental Table 1** Sensitivity analysis excluded people with a history of stroke, TIA, taking lipid-lowering or not taking antihypertensive medications (n=1255) | | | | |
| --- | --- | --- | --- | --- |
| ORs (95% CI) | Tertiles of PP | | | PP, per 1-SD mmHg |
|  | T1 | T2 | T3 |  |
| Model 1 | Ref. | 1.432 (1.081–1.896) | 2.504 (1.897–3.306) | 1.515 (1.343-1.709) |
| Model 2 | Ref. | 1.269 (0.949–1.696) | 2.063 (1.528-2.784) | 1.402 (1.232-1.596) |
| Model 1: unadjusted, Model 2: adjusted for a propensity score. OR odds ratio; CI confidence interval; PP pulse pressure. | | | | |
